# The Use of Rheumatic Disease Comorbidity Index for Predicting Clinical Response and Retention Rate in a Cohort of Rheumatoid Arthritis Patients Receiving Tumor Necrosis Factor Alpha Inhibitors

**DOI:** 10.1155/2019/6107217

**Published:** 2019-01-10

**Authors:** Martina Biggioggero, Federica Mesina, Ennio Giulio Favalli

**Affiliations:** ^1^Department of Rheumatology, Gaetano Pini Institute, Milan, Italy; ^2^Pfizer Innovative Health, I&I Medical Affairs, Rome, Italy

## Abstract

**Introduction:**

To retrospectively evaluate the impact of comorbidities on treatment choice, 12-month clinical response, and 24-month retention rate in a cohort of patients with rheumatoid arthritis (RA) treated with a first-line tumor necrosis factor alpha inhibitor (TNFi), by using for the first time the Rheumatic Disease Comorbidity Index (RDCI).

**Methods:**

The study population was extracted from a local registry of RA patients receiving adalimumab or etanercept as first-line biologics between January 2001 and December 2013. The prevalence of comorbidities was computed, and patients were stratified according to RDCI for evaluating the role of comorbidities on TNFi choice, concomitant methotrexate, clinical response (1-year DAS28-ESR remission and low disease activity [LDA] and EULAR good-moderate response), and the 24-month retention rate.

**Results:**

346 patients (172 adalimumab and 174 etanercept) were included. A significantly higher EULAR good/moderate response (*P = *0.020) and DAS28-ESR remission (*P = *0.003) were obtained according to RDCI (0, 1, 2, or ≥3). Lower RDCI (*P = *0.022), male sex (*P = *0.006), higher baseline DAS28-ESR (*P = *0.001), ETN (*P *< 0.001), and concomitant methotrexate (*P = *0.016) were predictors of EULAR good/moderate response. Elevated RDCI was a predictor of discontinuation of biologics (*P = *0.036), whereas treatment with etanercept (*P* < 0.001) and methotrexate (*P = *0.007) was associated with a lower risk of TNFi withdrawal.

**Conclusions:**

Multimorbidity, measured by RDCI, is a negative predictor of TNFi persistence on treatment and of achieving a good clinical response. The use of RDCI may be very useful for identifying patients with RA carrying those comorbid conditions associated with poor prognostic outcomes and for defining new treatment targets in multimorbid RA patients.

## 1. Introduction

Rheumatoid arthritis (RA) is an autoimmune disease characterized by a chronic articular inflammation leading to functional impairment and severe disability [1]. Moreover, beyond the joint involvement, RA is a systemic syndrome frequently associated with extra-articular manifestations, which can affect several organ systems [2]. In particular, more frequently than expected and in comparison to the general population, RA could be complicated by other conditions associated with the disease [3]. Some of these comorbidities are deeply interconnected with RA through shared pathogenic mechanisms leading to chronically active inflammation or to the increased presence of traditional risk factors, such as tobacco smoking [4–6]. Furthermore, comorbidities could be the consequence of chronic disease medications, especially corticosteroids [7]. The most common comorbidities observed in RA patients are cardiovascular and pulmonary diseases, infections, osteoporosis, depression, and malignancies [2, 8]. The impact of comorbidities on the management of RA is crucial and may be bidirectional. On the one hand, the elevated prevalence of comorbidities may contribute to affect the prognosis of the disease and compromise the life expectancy in RA patients [2]. Moreover, a poor clinical response has been demonstrated in RA patients carrying some comorbid conditions such as depression or fibromyalgia as a consequence of a worse perception by the patient of the disease, resulting in higher scores in the patient-reported components of disease activity indices [9, 10]. On the other hand, the presence of certain comorbidities may be a limitation to the usual application of optimal treatment strategies because of the existence of contraindication to the prescription of some drug classes. In particular, the history of recent malignancy or RA-related interstitial lung disease may significantly complicate the management of RA with synthetic or biologic disease-modifying antirheumatic drugs (bDMARDs) [11]. In recent decades, the introduction of targeted therapies and the application of the treat-to-target strategy have revolutionized the management of RA, reducing disability and improving the quality of life of patients [12].

As is standard, the efficacy and safety profile of TNFis was firstly evaluated in randomized controlled trials (RCTs), which enrolled very selected patients by the application of stringent criteria excluding patients affected by comorbidities potentially biasing the comparative analysis. Although RCTs are the best tool for comparing a new drug with the gold standard therapy, the enrolled study populations significantly differ from the majority of patients treated in daily life clinical practice, which are often ineligible for these trials [13, 14]. For those reasons, RCTs did not provide any information about the prevalence and the impact of the main comorbidities encountered in the management of RA patients receiving an anti-TNF agent. Thus, to date this topic has been addressed only by real-world observational studies, which demonstrated that comorbid conditions might be associated with a lower likelihood of achieving a good clinical response [15–20]. Accordingly, comorbidities have been included in the European League Against Rheumatism (EULAR) recommendation for the management of RA (overarching principle B) in the list of the factors to be considered in the application of treat-to-target strategy [21]. Despite the growing interest generated in the rheumatologic community about the identification and implications of comorbid conditions in patients with RA, specific methods for better quantifying this aspect of RA management have yet to be characterized, and the majority of the available analyses have been performed by using empiric scoring methods for measuring comorbidities.

In order to fill this gap, we performed an analysis of a local registry with the aim of retrospectively evaluating the impact of comorbidities on treatment choice, 12-month clinical response, and 24-month retention rate in a cohort of RA patients treated with a first-line subcutaneous TNFi, using for the first time the Rheumatic Disease Comorbidity Index (RDCI) as a baseline predictor of RA outcomes.

## 2. Materials and Methods

### 2.1. Selection of Study Population

Data from all RA patients aged ≥18 years fulfilling the American College of Rheumatology (ACR) 1987 revised criteria [22] and treated with bDMARDs in our Rheumatology Unit were collected in a local registry approved by the Gaetano Pini Institute Ethics Committee, including all patients who signed the informed consent for any subsequent retrospective analysis of their clinical data. The study population was composed of all RA patients receiving adalimumab or etanercept as a first-line biologic drug between January 2001 and December 2013. Exclusion criteria were previous therapy with a different bDMARD or the enrollment in an RCT.

All clinical information analyzed was reported as anonymous aggregate data, excluding any identifiable medical information. For all patients, the database includes demographic features (age, sex, and time since RA diagnosis); clinical parameters (C-reactive protein [CRP], erythrocyte sedimentation rate [ESR] level, RF positivity, disease activity score 28-ESR [DAS28-ESR], and Health Assessment Questionnaire Disability Index [HAQ-DI] score); the prevalence of common RA comorbidities at baseline (osteoporosis and osteoporotic fractures, hypertension, depression, cardiovascular disease, lung disease, fibromyalgia, autoimmune thyroid disease, dyslipidemia, gastrointestinal disorders, diabetes, neurological disorders, history of malignancy); and therapeutic data (biologic therapy and concomitant conventional synthetic DMARD [csDMARD]). All the mentioned disease and treatment follow-up data were collected at baseline and then every 6 months until December 2014. Treatments were administered as part of routine care in accordance with good clinical practice for RA; TNFis were prescribed according to their licensed regimen, and concomitant csDMARDs or corticosteroids were administered if ordered by the referring rheumatologist.

The study population was stratified according to RDCI score [23] (RDCI=0, 1, 2, or ≥3) to evaluate its role in the prediction of the impact of comorbidities on the baseline choice of treatment strategy and on drug survival and clinical efficacy.

### 2.2. Outcomes and Statistical Analysis

Descriptive statistics were used to calculate mean and standard deviation (SD), and median and interquartile range. Differences between subgroups according to RDCI score were analyzed by the Kruskal-Wallis nonparametric test for continuous variables and chi-square test for categorical variables.

The 2-year retention rate was computed by the Kaplan-Meier method and compared between subgroups by a stratified log-rank test. Moreover, a Cox proportional hazard model was developed to examine the role of RDCI and other baseline factors as predictors of TNFi persistence. Results are presented as hazard ratios (HRs) with 95% confidence intervals (95% CI).

Clinical response was evaluated as 12-month DAS28-ESR mean change from baseline, 12-month rate of patients achieving DAS28-ESR remission (DAS28-ESR <2.6) or low disease activity (LDA, DAS28-ESR ≥2.6 and <3.2), and 12-month proportion of patients achieving good/moderate response according to EULAR criteria. A multivariate logistic regression model was developed to examine the role of RDCI along with other baseline factors as potential predictor of achieving remission, LDA, and EULAR good/moderate response. Results are presented as odds ratio (ORs) with 95% CI. Both Cox proportional hazard model and logistic regression model included gender, RF positivity, TNFi agent, and concomitant methotrexate (MTX) as categorical variables, whereas age and disease duration at the beginning of TNFi therapy, DAS28-ESR, HAQ-DI, and RDCI were considered as continuous variables. Moreover, a separate univariate model was developed for analyzing the impact of each individual comorbidity on clinical response.

Statistical analyses were performed using SPSS statistical software, version 20.0 (SPSS Inc., Chicago, IL, USA).* P* values equal to or less than 0.05 were considered statistically significant.

### 2.3. Ethical Considerations

Collection and evaluation of the data were approved by the Gaetano Pini Institute Ethics Committee, all patients included in the study signed written informed consent for any subsequent retrospective analysis of their clinical data, and the study was conducted in accordance with the 1964 Declaration of Helsinki and its later amendments.

## 3. Results

### 3.1. Baseline Characteristics

A total of 346 patients (172 receiving adalimumab and 174 etanercept) were included in the analysis. The baseline demographic and clinical characteristics of the study population are reported in Table 1. Specifically, 282 of 346 patients (81.5%) were female, with mean (±SD) age of 53.45 ± 13.03 years, mean disease duration 11.48 ± 9.13 years, mean DAS28 5.29 ± 1.23, mean HAQ-DI 1.39 ± 0.56, and RF positive 75.1%. Among 157 (45.4% of the whole population) patients not receiving concomitant treatment with MTX (81 and 76 patients in the adalimumab and etanercept subgroups, respectively), 56 were concomitantly treated with another csDMARD (36 hydroxychloroquine, 4 cyclosporine, 15 leflunomide, and 1 sulfasalazine) and 101 received the TNFi as pure monotherapy. Among concomitant MTX users, 112 (33.9%) patients received low-dose (≤10 mg/weekly) and 77 (23.4%) high-dose MTX (≥12.5 mg weekly).

### 3.2. Prevalence of Comorbidities and Impact on Treatment Choice

The baseline prevalence of comorbid conditions is listed in Table 2. The most frequently reported comorbidities were osteoporosis (24.6%), hypertension (20.8%), depression (11.3%), and cardiovascular disorders (10.4%). The majority of patients (63.6%) carried at least one comorbidity (18.8%, 17.9%, and 26.9% with 1, 2, ≥3, respectively). No significant differences emerged in the comparison of baseline demographic and clinical characteristics of the 4 subgroups according to the prevalence of comorbidities (Table 1), with the only exception of a progressively increasing mean age by increasing RDCI score (*P = *0.001). The overall mean (±SD) RDCI score was 0.71 ± 1.04, with no difference according to the prescribed anti-TNF agent (adalimumab 0.72 ± 0.96 and etanercept 0.71 ± 1.12). No clear trend of decreasing MTX prescription by RDCI score increasing was found (*P = *0.09) and the proportion of patients with RDCI ≥1 was similar in concomitant MTX users compared with patients receiving the TNFi without MTX (39.7% vs. 42.7%, respectively;* P = *0.585). Similarly, RDCI score was not associated with a preferential prescription of an individual TNFi (*P = *0.306), and the rate of RDCI ≥ 1 was similar in patients treated with adalimumab compared with etanercept (44.2% vs. 37.9%, respectively;* P = *0.275). As detailed in Table 2, no individual comorbidity was significantly correlated with the prescription of concomitant MTX or with the choice between the two TNFis.

### 3.3. Predictive Role of RDCI on Clinical Response

The 12-month clinical response rates in the whole population were 58.9%, 12.1%, and 30.6% for EULAR good/moderate response, DAS28-ESR LDA, and DAS28-ESR remission, respectively. The overall mean change from baseline of DAS28-ESR was 1.65 ± 3.08 (*P* < 0.0001 vs. baseline).

Compared with patients carrying at least one comorbidity, the lack of comorbid disorders was not associated with a different 12-month EULAR good-moderate response (54.2% vs. 62.3%, respectively;* P = *0.149), DAS28-ESR LDA (12.7% vs. 11.3%, respectively;* P = *0.740), or DAS28-ESR remission (28.2% vs. 32.4%, respectively;* P = *0.477). However, according to the multivariate logistic regression analysis, a lower RDCI score was a predictor of achieving 12-month EULAR good-moderate response (OR 0.746, 95% CI 0.580-0.960,* P = *0.022), but not DAS28-ESR LDA (OR 1.016, 95% CI 0.719-1.434,* P = *0.930) or remission (OR 0.776, 95% CI 0.579-1.040,* P = *0.090). Moreover, male sex, higher baseline DAS28-ESR, treatment with etanercept, and concomitant MTX were also all associated with EULAR good-moderate response, while older age, male sex, and higher baseline DAS28-ESR were correlated with DAS28-ESR remission (Table 3). In the univariate analysis for the likelihood of achieving an EULAR response, LDA, or remission, no individual comorbidity was identified as a predictor of clinical response according to all of the considered outcomes.

### 3.4. Predictive Role of RDCI on TNFi Retention Rate

**Figure 1:**
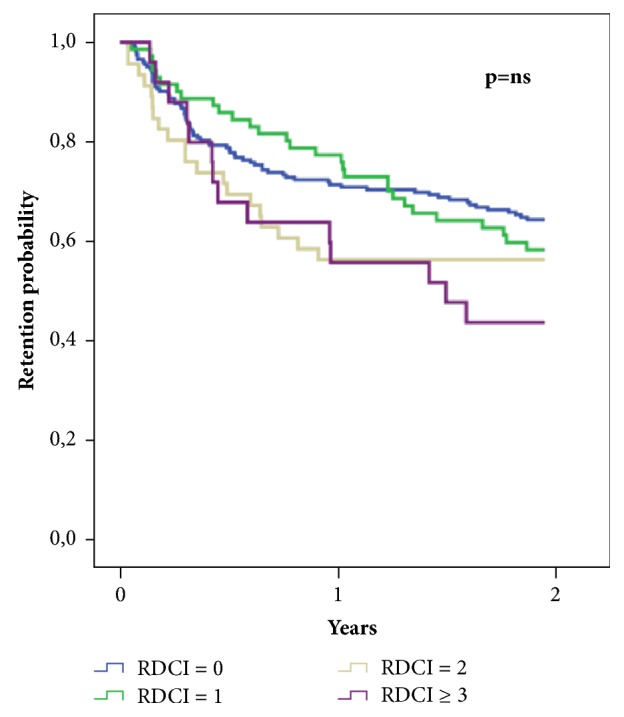
Unadjusted two-year retention rate of tumor necrosis factor inhibitors (TNFis) according to baseline Rheumatic Disease Comorbidity Index (RDCI).

The overall 24-month retention rate in the whole population was 61%. 74 (54.8%) patients stopped first-course TNFi because of inefficacy (39 [52.7%] due to primary lack of response) and 61 [45.2%] because of adverse events). No significant differences were found in the crude retention rate analysis according to baseline RDCI score, as illustrated in Figure 1.

However, the application of a Cox model considering baseline characteristics showed increasing RDCI as a predictor of biologic drug discontinuation (HR 1.186, 95% CI 1.011-1.390;* P = *0.036), whereas treatment with etanercept (HR 0.493, 95% CI 0.343-0.707;* P* < 0.001) and concomitant MTX (HR 0.622, 95% CI 0.440-0.877;* P = *0.007) were both associated with a lower risk of TNFi withdrawal. The detailed results of the Cox regression analysis are reported in Table 4.

## 4. Discussion

Our observational retrospective analysis confirmed that comorbidities might affect RA outcomes in TNFi-treated patients, with an apparently greater effect on long-term retention rate than on short-term clinical response. Increasing RDCI score is a predictor of both higher 2-year TNFi discontinuation and lower likelihood of achieving a 1-year EULAR good-moderate response, suggesting its potential use in clinical practice for the screening of baseline comorbidity status in patients intended to receive a TNFi.

In recent years, there has been a growing interest in how the presence of comorbidities could affect the management and prognosis of RA patients. The EULAR initiative developed a number of proposed points to consider for reporting, screening, and preventing selected comorbidities based on their frequency and severity in chronic inflammatory rheumatic disease [8]. The most relevant data about the prevalence of comorbidities in RA were reported by the international cross-sectional COMORA (COMOrbidities in Rheumatoid Arthritis) study [2], which collected between 2011 and 2012 information from 17 participating countries finding that the most prevalent comorbid conditions were past or current depression (15%), gastrointestinal ulcers (10.8%), and pulmonary diseases (11%). These findings differ from the ones observed in our population, where osteoporosis, hypertension, depression, and cardiovascular diseases were the most frequently associated disorders. The reasons for this apparent discrepancy may lie in the wide variation among participant countries observed in the COMORA study and in the different baseline characteristics of enrolled patients. In fact, while Dougados and colleagues included a broad population treated with both synthetic and biologic DMARDs, we restricted recruitment only to subjects treated with TNFis, thus excluding those patients carrying comorbidities such as malignancies, which represent a contraindication to the use of bDMARDs. A cross-sectional study from Sweden recently confirmed this hypothesis by reporting a significantly higher prevalence of comorbid conditions in patients who received synthetic compared with biologic DMARDs over a 5-year follow-up period [24]. In particular, this study found older age, cerebrovascular and lung diseases, heart failure, depression, and history of malignancies to be predictors of a treatment strategy excluding bDMARDs.

Older age has been reported to be associated with an increased number of comorbidities in RA patients [25]. These data have been confirmed by our analysis, clearly showing a statistically significant trend in RDCI score increment by increasing age. Nevertheless, in our population, both age and presence/number of comorbidities did not appear to influence treatment decision making in the choice between etanercept and adalimumab and between TNFi monotherapy or concomitant MTX therapy.

Our study provides the firstly published data about the association of comorbidities with treatment strategy within the TNFi class, since previous studies have mainly addressed the role of comorbidities as a driver of treatment choice between bDMARDs with different mechanisms of action [26, 27]. Regarding the use of concomitant MTX, we have previously demonstrated that combination therapy with TNFis is associated with a lower discontinuation rate because of adverse events compared with TNFi monotherapy [28]. Thus, it may be reasonable that the presence of comorbidities has not been considered a contraindication to the concomitant use of MTX, given the overall favorable safety profile of combination strategy [29].

Several papers have reported the relationship between comorbidities and a reduced probability of achieving a clinical response. A large open-label study conducted in 6,610 RA patients treated with adalimumab described a higher likelihood of achieving DAS28 remission (OR 0.86) in subjects carrying one or no comorbidity compared with those with more than one [15]. Similarly, a multinational cross-sectional study including 5,848 RA patients demonstrated a significant association (OR 0.75, 95% CI 0.68-0.83) between the number of comorbidities and the Clinical Disease Activity Index (CDAI) [30]. More recently data from the CORRONA registry further confirmed that fewer comorbidities, together with higher baseline CDAI, were associated with a greater improvement in CDAI and a higher probability of CDAI remission [18]. However, those studies were conducted over a short-term follow-up period and did not evaluate the impact of multimorbidity on long-term treatment outcomes. Moreover, the burden of comorbidities has been measured only as the crude number of comorbid conditions rather than by the use of a specific comorbidity index. On the other hand, Nakajima et al. reported in a large Japanese cohort the impact of increasing values of the Charlson Comorbidity Index (CCI) [31] on clinical response measured by DAS28 and physical ability according to HAQ score, demonstrating a significant difference only in the comparison between patients with CCI=0 and those with CCI ≥1 [20]. As another example of the use of a comorbidity index for measuring the role of comorbidities in clinical response, Radner et al. [32] recently published an analysis of the BRASS cohort by using the counted multimorbidity index (cMMI), based on the impact of 40 different comorbidities on health-related quality of life [33]. In this study, the OR associated with each additional morbidity in the cMMI was 0.72 (95% CI 0.55, 0.97) for remission and 0.81 (95% CI 0.70, 0.94) for LDA.

Compared with those studies, our analysis was performed over a 2-year period by using for the first time the RDCI score [23], which is specifically developed for evaluating the prevalence of comorbidities in patients with rheumatologic disorders rather than in the general population. This tool may provide a more accurate measure of the overall comorbidity burden of RA than other widely used indices such as the CCI or the Elixhauser Comorbidity Score (ECS) [34]. In fact, both CCI and ECS were created to predict death or hospital charges in samples of hospitalized patients but have been subsequently applied in different clinical situations extending beyond their intended scope. Moreover, the exclusion of relevant comorbidities for RA, such as hypertension, osteoporosis, and depression, makes CCI and ECS incomplete tools for a comprehensive evaluation of comorbid disorders of rheumatoid disease. Finally, compared with the only other index validated for rheumatic diseases, the cMMI [33], RDCI seems to perform better in RA patients as reported by a recent nonsystematic literature review about this topic [35]. Our data demonstrated the efficacy of RDCI score in predicting 2-year retention rate and 1-year clinical response, with a statistical significance for EULAR good-moderate response and a clear trend for remission. No individual comorbid condition was found to be associated with RA outcomes, suggesting that a global score measuring the overall comorbidity burden rather than the presence of a single associated disease may be a more accurate predictor of clinical response and drug persistence.

In addition to multimorbidity, we identified other predictors of TNFi persistence on treatment, specifically the use of etanercept rather than adalimumab and the concomitant therapy with MTX, which is in line with the published literature [36–38].

The main limitation of our work lies in the fact that the evaluated database was not explicitly designed for the study purpose. Thus our retrospective analysis was conducted on a limited cohort of patients, which cannot be considered as entirely representative of whole RA population. However, the selection of a homogeneous group of patients treated with the same drug class allowed the avoidance of potential differences related to the use of synthetic or biologic DMARDs, providing clinically relevant results for the daily use of TNFis.

## 5. Conclusions

In conclusion, multimorbidity measured by RDCI is a negative predictor of TNFi persistence on treatment and of achieving a good clinical response. The use of a specific comorbidity score such as RDCI may be very useful for identifying RA patients carrying those comorbid conditions associated with poor prognostic outcomes and for defining new treatment targets in multimorbid RA patients.

## Figures and Tables

**Table 1:** Baseline characteristics of the study population.

	Overall	RDCI=0	RDCI=1	RDCI=2	RDCI≥3	*P*
	*n* = 346	*n* = 204	*n* = 71	*n* = 46	*n* = 25	
Mean age ± SD, years	53.45 ± 13.03	51.59±13.59	53.49±12.2	57.56±11.33	60.91±9.2	0.001*∗*
Female, *n *(%)	282 (81.5)	169 (82.8)	58 (81.7)	38 (82.6)	17 (68)	0.347*∗∗*
Mean disease duration ± SD, years	11.48 ± 9.13	11.37±8.91	11.59±10.92	11.43±8.23	12.15±7.08	0.515*∗*
Mean DAS28-ESR ± SD	5.29 ± 1.23	5.27±1.22	5.17±1.33	5.48±1.18	5.40±1.06	0.842*∗*
Mean HAQ score ± SD	1.39 ± 0.56	1.34±0.55	1.38±0.53	1.52±0.60	1.54±0.68	0.134*∗*
Positive RF, *n* (%)	260 (75.1)	151 (74)	51 (71.8)	37 (80.4)	21 (84)	0.514*∗∗*
Concomitant MTX, n (%)	189 (54.6)	114 (55.9)	36 (50.7)	22 (47.8)	17 (68)	0.099*∗∗*
Low-dose, *n* (%)	112 (33.9)	65 (57.0)	17 (47.2)	17 (77.3)	13 (76.5)	
High-dose, *n* (%)	77 (23.4)	49 (43.0)	19 (52.8)	5 (22.7)	4 (23.5)	
bDMARD, *n* (%)						0.306*∗∗*
Etanercept	174 (50.3)	108 (53.6)	31 (43.6)	20 (43.4)	15 (60.0)	
Adalimumab	172 (49.7)	96 (46.4)	40 (56.4)	26 (56.6)	10 (40.0)	

bDMARD: Biologic Disease Modifying Antirheumatic Drugs; DAS28-ESR: Disease Activity Score 28-Erythrocyte Sedimentation Rate; HAQ: Health Assessment Questionnaire; MTX: Methotrexate; RDCI: Rheumatic Disease Comorbidity Index; RF: Rheumatoid Factor; SD: Standard Deviation. *∗*Kruskal-Wallis test; *∗∗* chi-squared test.

**Table 2:** Baseline prevalence of comorbidities.

Comorbidity, n (%)	Overall *n* = 346	TNFis			Concomitant MTX		
		ADA	ETN	*P*	No	Yes	*P*
		*n* = 172	*n* = 174		*n* = 157	*n = *189	
Osteoporosis	85 (24.6)	41 (23.8)	44 (25.3)	0.803	45 (28.6)	40 (21.1)	0.132
Hypertension	72 (20.8)	33 (19.2)	39 (22.4)	0.509	37 (23.6)	35 (18.5)	0.288
Depression	39 (11.3)	25 (14.5)	14 (8)	0.063	13 (8.3)	26 (13.7)	0.126
Cardiovascular disease	36 (10.4)	17 (9.9)	19 (10.9)	0.861	15 (9.6)	21 (11.1)	0.725
Lung disease	26 (7.5)	11 (6.4)	15 (8.6)	0.542	12 (7.6)	14 (7.4)	1
Fibromyalgia	25 (7.2)	12 (7)	13 (7.5)	1	8 (5.1)	17 (9)	0.211
Autoimmune thyroid disease	19 (5.5)	8 (4.6)	11 (6.3)	0.638	12 (7.6)	7 (3.7)	0.154
Dyslipidemia	19 (5.5)	5 (2.9)	14 (8)	0.057	7 (4.4)	12 (6.3)	0.486
Diabetes	12 (3.5)	6 (3.5)	6 (3.4)	1	4 (2.5)	8 (4.2)	0.558
Gastrointestinal disorders	10 (2.9)	5 (2.9)	5 (2.9)	1	6 (3.8)	4 (2.1)	0.522
Osteoporotic fractures	8 (2.3)	5 (2.9)	3 (1.7)	0.501	6 (3.8)	2 (1.1)	0.148
Neurological disorders	6 (1.7)	1 (0.6)	5 (2.9)	0.215	1 (0.6)	5 (2.6)	0.227
History of malignancy	1 (0.3)	0 (0)	1 (0.6)	1	0 (0)	1 (0.5)	1

ADA: adalimumab; ETN: etanercept; MTX: methotrexate; TNFis: tumor necrosis factor alpha inhibitors.

**Table 3:** The role of RDCI and other baseline factors as predictors of 12-month EULAR good/moderate response and remission or low disease activity.

	EULAR good/moderate response			Remission			Low disease activity		
	OR	95% CI	*P*	OR	95% CI	*P*	OR	95% CI	*P*
Age, years	0.992	0.972-1.012	0.435	0.975	0.954-0.997	0.027	1.001	0.972-1.030	0.954
Sex	0.404	0.211-0.776	0.006	0.460	0.245-0.862	0.015	0.512	0.240-1.094	0.084
Disease duration, years	0.992	0.966-1.019	0.553	0.986	0.956-1.018	0.387	1.017	0.980-1.055	0.364
DAS28-ESR	1.540	1.191-1.990	0.001	0.541	0.404-0.724	<0.0001	0.955	0.674-1.351	0.793
HAQ	1.013	0.582-1.762	0.965	1.200	0.654-2.200	0.557	0.954	0.448-2.031	0.903
RF	1.749	0.982-3.114	0.058	1.739	0.988-3.063	0.055	1.055	0.489-2.278	0.891
Concomitant MTX	0.561	0.351-0.897	0.016	0.631	0.374-1.062	0.083	0.717	0.365-1.410	0.335
bDMARD (ETN vs ADA)	2.523	1.569-4.056	<0.001	1.623	0.966-2.729	0.067	1.557	0.792-3.062	0.199
RDCI	0.746	0.580-0.960	0.022	0.776	0.579-1.040	0.090	1.016	0.719-1.434	0.930

ADA: Adalimumab; DAS28-ESR: Disease Activity Score 28-Erythrocyte Sedimentation Rate; ETN: Etanercept; EULAR: European League Against Rheumatism; HAQ: Health Assessment Questionnaire; MTX: Methotrexate; OR: Odds Ratio; RDCI: Rheumatic Disease Comorbidity Index; RF: Rheumatoid Factor.

**Table 4:** The role of RDCI and other baseline factors as predictors of TNFi persistence.

	HR	95% CI	*P*
Age, years	1.005	0.990-1.019	0.523
Sex	1.090	0.684-1.738	0.716
Disease duration, years	0.999	0.979-1.018	0.897
DAS28-ESR	1.122	0.934-1.348	0.218
HAQ	0.926	0.608-1.410	0.721
RF	1.198	0.796-1.803	0.386
Concomitant MTX	0.622	0.440-0.877	0.007
bDMARD (ETN vs ADA)	0.493	0.343-0.707	<0.001
RDCI	1.186	1.011-1.390	0.036

HR: hazard ratio; CI: confidence intervals; DAS28-ESR: disease activity score 28- erythrocyte sedimentation rate; HAQ: health assessment questionnaire; RF: rheumatoid factor; MTX: methotrexate; bDMARD: biologic disease modifying anti-rheumatic drugs; ETN: etanercept; ADA: adalimumab; RDCI: rheumatic disease comorbidity index.

## Data Availability

The data sets used to support the current study are available from the corresponding author on reasonable request.
